# SR-CycleGAN: super-resolution of clinical CT to micro-CT level with multi-modality super-resolution loss

**DOI:** 10.1117/1.JMI.9.2.024003

**Published:** 2022-04-05

**Authors:** Tong Zheng, Hirohisa Oda, Yuichiro Hayashi, Takayasu Moriya, Shota Nakamura, Masaki Mori, Hirotsugu Takabatake, Hiroshi Natori, Masahiro Oda, Kensaku Mori

**Affiliations:** a Nagoya University, Graduate School of Informatics, Furo-cho, Chikusa-ku, Nagoya, Japan; b Nagoya University, Graduate School of Medicine, Nagoya, Japan; c Sapporo-Kosei General Hospital, Sapporo, Japan; d Sapporo Minami-Sanjo Hospital, Sapporo, Japan; e Keiwakai Nishioka Hospital, Sapporo, Japan; f Nagoya University, Information Strategy Office, Information and Communications, Nagoya, Japan; g Nagoya University, Information Technology Center, Nagoya, Japan; h National Institute of Informatics, Research Center of Medical BigData, Tokyo, Japan

**Keywords:** unpaired super-resolution, detailed anatomical information, inter-modality translation, lung micro-anatomy

## Abstract

**Purpose:**

We propose a super-resolution (SR) method, named SR-CycleGAN, for SR of clinical computed tomography (CT) images to the micro-focus x-ray CT CT (μCT) level. Due to the resolution limitations of clinical CT (about $500\times 500\times 500\;{\mu m}^{3}/\text{voxel}$), it is challenging to obtain enough pathological information. On the other hand, μCT scanning allows the imaging of lung specimens with significantly higher resolution (about $50\times 50\times 50\;{\mu m}^{3}/\text{voxel}$ or higher), which allows us to obtain and analyze detailed anatomical information. As a way to obtain detailed information such as cancer invasion and bronchioles from preoperative clinical CT images of lung cancer patients, the SR of clinical CT images to the μCT level is desired.

**Approach:**

Typical SR methods require aligned pairs of low-resolution (LR) and high-resolution images for training, but it is infeasible to obtain precisely aligned paired clinical CT and μCT images. To solve this problem, we propose an unpaired SR approach that can perform SR on clinical CT to the μCT level. We modify a conventional image-to-image translation network named CycleGAN to an inter-modality translation network named SR-CycleGAN. The modifications consist of three parts: (1) an innovative loss function named multi-modality super-resolution loss, (2) optimized SR network structures for enlarging the input LR image to $2^{k}$-times by width and height to obtain the SR output, and (3) sub-pixel shuffling layers for reducing computing time.

**Results:**

Experimental results demonstrated that our method successfully performed SR of lung clinical CT images. SSIM and PSNR scores of our method were 0.54 and 17.71, higher than the conventional CycleGAN’s scores of 0.05 and 13.64, respectively.

**Conclusions:**

The proposed SR-CycleGAN is usable for the SR of a lung clinical CT into μCT scale, while conventional CycleGAN output images with low qualitative and quantitative values. More lung micro-anatomy information could be observed to aid diagnosis, such as the shape of bronchioles walls.

## 1 Introduction

Currently, lung cancer is the most common cancer among men,^1^ and the most common cause of cancer death worldwide.^2^ In 2020, following the level of female breast cancer diagnoses, an estimated 2.2 million cases of lung cancer were newly diagnosed (11.4% of total new cancer cases). Lung cancer remains the leading cause of cancer death, with an estimated 1.8 million deaths (18% of total cancer deaths).^3^ Most lung cancers are not found in their early stage, and clinical computed tomography [clinical CT (we use the term “clinical CT image” for CT images that are conventionally taken at hospitals. We use the term “CT volumes” for volumetric images acquired by CT scanning, and we use the term “CT images” for two-dimensioanl (2D) images cropped from CT volumes.)] by volumetric image scanning is offered to patients considered to be at high risk of contracting the disease.^4^ Clinical CT of lung cancer patients is also used for planning surgery, radiotherapy, and chemotherapy.^5^ Clinical CT of lung cancer patients provides more detailed images than chest x-rays and is better at finding small abnormal areas in the lungs.^6^ However, the resolution of clinical CT is still not high enough to observe some micro anatomical structures. We cannot observe enough pathological informations, such as the invasion of cancer, and thin bronchioles, from clinical CT due to its limited resolution (about $500\times 500\times 500\;\mu m^{3}/\text{voxel}$).^7^ To acquire more detailed pathological information for preoperative diagnosis, it is important to enhance the resolution of clinical CT images.

**Fig. 1:**
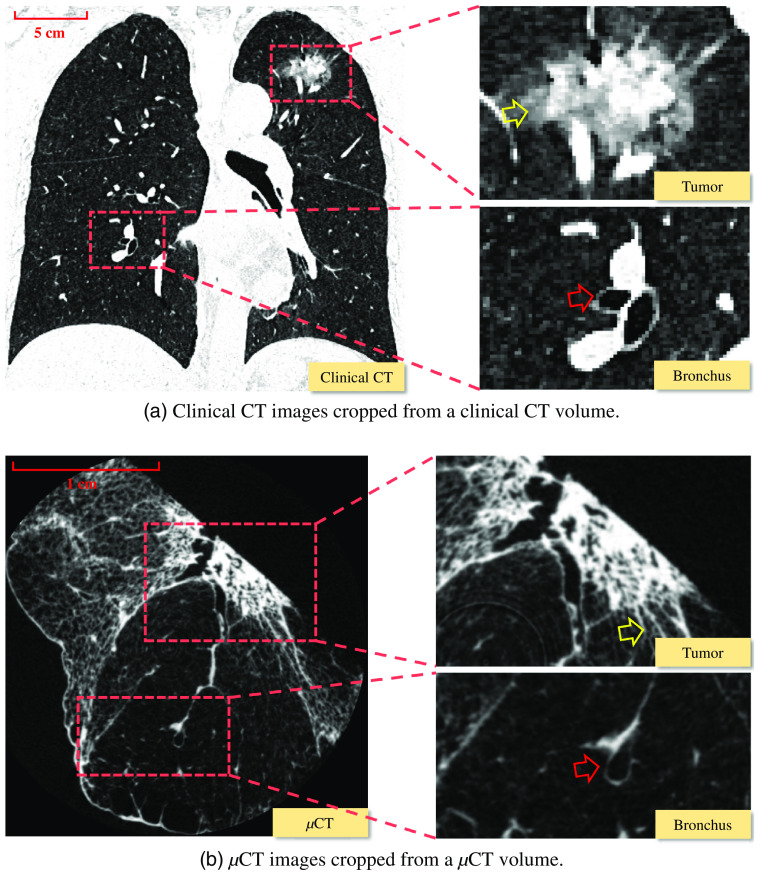
Comparison of clinical CT and μCT. In (a), the surrounding of the tumor (yellow arrows) and edge of bronchus (red arrows) are jagged. We can obtain from (b) about the tumor’s invasion (tumor cells to disrupt the basement membrane and invade other tissues,^10^ pointed by yellow arrows) and the apparent edge of the bronchus (red arrows). The resolution of (a) and (b) is totally different, as shown by the red scale line.

Micro-focus x-ray CT (μCT) is another CT modality, and it can take images of a much higher resolution than those by CT. Although μCT cannot scan living human bodies,^8^ it can scan small targets, e.g., a surgically dissected human lung, the entire body of a mouse, or a rabbit heart. Isotropic resolution of μCT volumes is typically $50\times 50\times 50\;\mu m^{3}/\text{voxel}$ or higher. μCT volumes obtained by μCT scanning of resected lung cancer specimens can capture their detailed and surrounding anatomical structures.^9^ A comparison of clinical CT images with μCT images is shown in Fig. 1. We can clearly observe tumor’s outline and bronchus from μCT, while tumor outline and the bronchus are jagged in clinical CT.

If we could enhance the resolution of lung cancer patients’ clinical CT images, we would be able to observe detailed anatomical structures, such as thin bronchioles, and then use the resolution-enhanced clinical CT to guide surgeries and treatment plans for lung cancer. Furthermore, a better resolution may substantially improve automatic detection and image segmentation results.^11^ Super-resolution (SR) is a term for a set of methods of enhancing the resolution of video or images.^12^ Our goal is to perform SR of the clinical CT images of lung cancer patients.

Deep learning (DL)-based methods for medical image analysis have become active in recent years.^13^ DL-based methods have achieved state-of-the-art (SOTA) accuracy^14^^–^^18^ over traditional methods in segmentation. DL-based methods also achieved SOTA in medical image denoising.^19^^,^^20^ Following this trend, we also use DL-based methods for performing SR in this paper.

Previous SR methods based on DL^21^^–^^25^ commonly needed aligned pairs of low-resolution (LR) and high-resolution (HR) images to train a fully convolutional network^26^ for SR. Dong et al.^21^ proposed a deep neural network-based SR method for single-image SR. Ledig et al.^22^ proposed a generative adversarial network (GAN) for photorealistic SR. Lim et al.^23^ proposed an enhanced deep residual network^27^ for SR. Haris et al.^24^ proposed a network that exploits iterative up- and down-sampling layers for SR. Wang et al.^25^ proposed a dual-stream network for SR. There are also several approaches to the SR of CT images.^28^^–^^30^ Yu et al.^28^ proposed a single-slice and multi-slice SR method for CT images. Georgescu et al.^30^ proposed a two-stage network for the SR of CT and MRI images. However, a common disadvantage of the above methods^21^^–^^25^^,^^28^^–^^30^ is that they require paired LR-HR images for training. LR images are acquired by downsampling the HR images using interpolation algorithms such as bicubic interpolation.^31^

It is difficult to perform the SR of lung clinical CT images using these previous methods. Given a clinical CT image (regarded as LR image here) with a resolution of around $500\times 500\times 500\;\mu m^{3}/\text{voxel}$, we cannot acquire its corresponding HR image because it is difficult to scan a living human body at a higher resolution. On the other hand, we can obtain μCT images having a micro-level resolution by scanning resected lung specimens. We can use μCT images of lung specimens to guide the SR of lung clinical CT images. Since lung clinical CT and μCT are acquired from different imaging devices, image registration of lung clinical CT and μCT images is needed to obtain paired LR (clinical CT)-HR (μCT) images of the lung. However, registration between clinical CT and μCT is challenging because the shape and inflation status of lung specimens in μCT images are very different from those of a living lung. Therefore, an unsupervised method that does not require pairs of clinical CT and μCT images is desired. However, there are very few unsupervised SR methods that do not require paired LR and HR images. Yuan et al.^32^ proposed an unsupervised method for single-image SR. However, this method is improper for processing medical images due to its unstable training process and excessive training time. Ravì et al.^33^ proposed an unsupervised SR method for endomicroscopy; however, this method requires certain hardware parameters for the endomicroscopy imaging device. Accordingly, there is demand for stable, time efficient, and highly versatile unsupervised SR method.

This paper proposes SR-CycleGAN, an unsupervised SR method that does not require paired LR-HR images to perform the SR of lung clinical CT images. First, we introduce a novel loss function named multi-modality super-resolution (MMSR) loss for preventing intensity variation of an SR image from the original domain (clinical CT) into the HR domain (μCT). Second, we design an optimal and time-saving network structure for SR. To prove our method’s effectiveness, we built a clinical-μCT database for our experiments and evaluated our method using this database. To the best of our knowledge, our method is the first approach to perform the SR of clinical CT using μCT.

The contributions of our method are: (1) a novel loss function named MMSR loss for cross-modality SR from clinical CT to μCT scale, (2) a specially designed SR network structure for shortening training time and enhancing accuracy, and (3) a newly built clinical CT−μCT dataset for verifying the feasibility of our proposed cross-modality SR method. Our code is available at https://github.com/zhuofeng/SR-cycleGAN.

## 2 Method

### 2.1 Overview

We propose an unsupervised method for performing the SR of clinical CT to the μCT-scale, using unpaired clinical CT−μCT images for training. We call our method SR-CycleGAN, since the structure of SR-CycleGAN is based on CycleGAN. The novelty of SR-CycleGAN consists of three aspects: (1) a network for SR, where the image-to-image translation networks of conventional CycleGAN were replaced by SR networks. The output SR image size is $2^{k}$-times (k∈N) larger than the input LR image. (2) A loss function named MMSR loss, which ensures that the output SR image has the same structure as that of the input LR image. (3) An optimized network structure for reducing training time and achieving better quantitative/qualitative results.

For training, our method requires clinical CT images and μCT images. Inputs of the network are 2D CT images (LR images) cropped from clinical CT volumes. Outputs are corresponding SR images. It is noteworthy that the height and width of SR images are $2^{k}$-times (k∈N) larger than those of the LR image.

### 2.2 Conventional CycleGAN

This section explains conventional CycleGAN to better understand our SR-CycleGAN. CycleGAN^34^ is an unsupervised image-to-image translation method based on deep generative models. It can learn to translate an image from a source domain X to a target domain Y in the absence of paired examples. The mathematical idea of CycleGAN is to obtain a generator $G_{1}$: X→Y and another generator $G_{2}$: Y→X. At the training stage of CycleGAN, the generators $G_{1}$ and $G_{2}$ are trained simultaneously, and a loss named cycle-consistency loss is adopted to maintain cycle-consistency $G_{2}(G_{1}(x))\approx x$ and $G_{1}(G_{2}(y))\approx y$. Here, x and y are the images from domain X and domain Y, respectively. The cycle-consistency loss is formulated as

$$L_{\mathrm{cyc}}(x,G_{2}(G_{1}(x))),y,G_{1}(G_{2}(y)))=E_{x∼X,y∼Y}[{\left\|x,G_{2}(G_{1}(x)))\right\|}_{2}^{2}+{\left\|y,G_{1}(G_{2}(y))\right\|}_{2}^{2}],$$ (1)

where ${\left\|\cdot \right\|}_{2}^{2}$ is the $l^{2}$-norm. Furthermore, to generate more realistic images, a CNN-based discriminator $D_{1}$ is used to distinguish generated images $G_{1}(x)$ and real images y. In addition, another generator $D_{2}$ is used to distinguish generated images $G_{2}(y)$ and real images x. Accordingly, generators $G_{1}$ and $G_{2}$ are trained to fool the discriminators $D_{1}$ and $D_{2}$. Moreover, $D_{1}$ and $D_{2}$ will help generators $G_{1}$ and $G_{2}$ to generate images that are closer to the target domain. Achieving this objective of generating more realistic images involves loss terms named adversarial losses. The adversarial losses are formulated as

$$L_{\mathrm{GAN}}(G_{1}(x),y)=E_{x∼X,y∼Y}[\log\;D_{1}(y)+(1-\log\;D_{1}(G_{1}(x)))],\;L_{\mathrm{GAN}}(G_{2}(y),x)=E_{x∼X,y∼Y}[\log\;D_{2}(x)+(1-\log\;D_{2}(G_{2}(y)))].$$ (2)

The combination of adversarial losses and cycle-consistency loss is used for the unpaired image-to-image translation in CycleGAN.

### 2.3 SR-CycleGAN

**Fig. 2:**
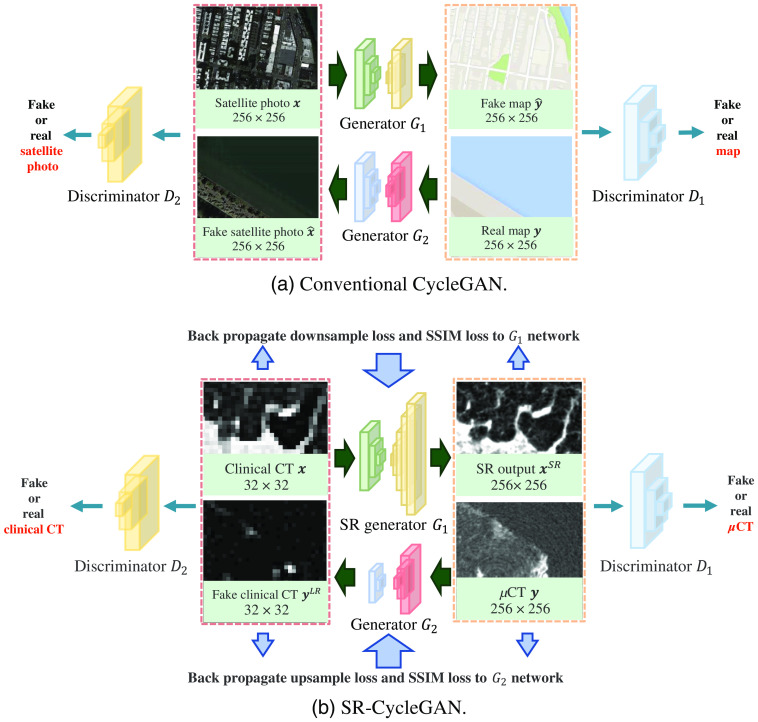
Structure comparison of (a) conventional CycleGAN and (b) SR-CycleGAN (our method). Conventional CycleGAN is an image-to-image translation network, where both its input and output are 256×256  pixels. Our method is an SR network. Its input size is 32×32  pixels, where its output size is 256×256  pixels.

The conventional CycleGAN is not designed for SR. Since CycleGAN is an image-to-image translation network, output and input images are of the same size. However, in performing the SR of a given image, the output image’s size is larger than the input image, since the output image’s resolution is higher than that of the input. Furthermore, CycleGAN faces problems such as providing diverse outputs.^35^ In the SR of medical images, we desire an output image that has the same anatomical structures as the input image. The SR result of a bronchus should still have the shape of a bronchus. Due to such constraints, we propose an SR network based on CycleGAN, and we named our method SR-CycleGAN. The structures of CycleGAN and SR-CycleGAN are shown in Fig. 2. Here, the input size and output size of CycleGAN are the same, but the output size is larger than the input in SR-CycleGAN.

#### 2.3.1 Network structure of SR-CycleGAN

**Fig. 3:**
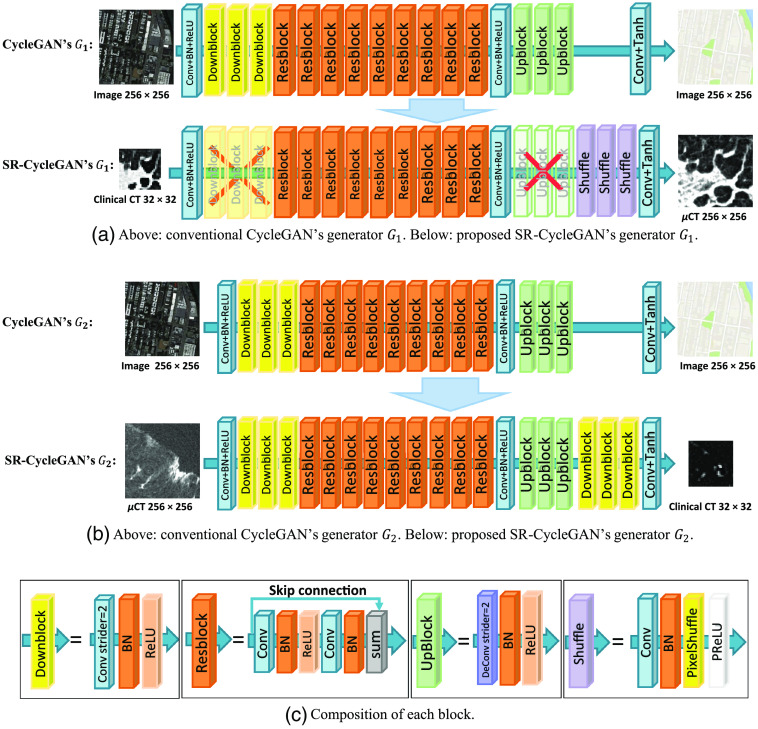
Modification from CycleGAN to SR-CycleGAN. The modifications of $G_{1}$ are as follows. (1) Removal of downblocks to maintain spatial information of the input image as shown in (a). (2) Removal of upblocks because feature maps no longer need them for scaling up as shown in (b). (3) Addition of sub-pixel shuffling layers at the end of the network for scaling up feature maps to the SR image. $G_{2}$ is a generator that shrinks an input image of 256×256  pixels into an image of 32×32  pixels. We added three downsample blocks (downblocks) to generator $G_{2}$. The specific structure of each block is shown in (c).

The specific network structure of SR-CycleGAN is shown in Fig. 3. As shown in Fig. 3(a), we modified conventional CycleGAN’s image-to-image translation neural network (generator) $G_{1}$ to an SR neural network by removing downblocks/upblocks (definitions of downblocks/upblocks are given in Fig. 3) and adding pixel-shuffling layers. In conventional CycleGAN, the input and output of $G_{1}$ are of the same size. We input an image with a size of n×n pixels into $G_{1}$ of CycleGAN. Then we obtained the same-sized image of n×n pixels as output. On the other hand, by inputting an image with a size of n×n pixels into $G_{1}$ of SR-CycleGAN, we obtained an image of $2^{k}n\times 2^{k}n$ (k∈N) pixels as output. The original network structure of generator $G_{1}$ has three “downblocks” at the network’s beginning, as shown in Fig. 3. Each downblock contains a convolution layer that scales down the image to 1/2 of its original size, following a batch normalization layer and an activation layer. If we input an image of 32×32  pixels into three downblocks, we would obtain feature maps of 4×4  pixels. Such small feature maps would wash away the spatial features of the given image. Therefore, we remove the downblocks of the generator $G_{1}$. Upblocks consist of deconvolution layers that scale up the feature maps to their original size in generator $G_{1}$ of CycleGAN. Since we remove the downblocks in SR-CycleGAN, the feature maps are no longer scaled-down, and thus we also remove the upblocks in SR-CycleGAN. Finally, SR-CycleGAN is an SR network. Thus, we need to scale up feature maps at the end of the network to obtain the SR image. Use of a sub-pixel shuffling layer has been proven to reduce computational complexity, save computing time, and perform significantly better than using a deconvolution layer in SR operation.^36^ Therefore, we add sub-pixel shuffling layers at the end of the network for scaling up feature maps to obtain the SR image as shown in Fig. 3(a).

In SR-CycleGAN, generator $G_{2}$ is an inverse function of generator $G_{1}$. Since generator $G_{1}$ scales up an input image to an SR image, we modified the generator $G_{2}$ to scale down an HR image to an LR image. In conventional CycleGAN, an image with a size of $2^{k}n\times 2^{k}n$ (k∈N) pixels is input into $G_{2}$, and an image of the same size is produced as output. On the other hand, in generator $G_{2}$ of SR-CycleGAN, we obtain an image of n×n as output from an input image of size $2^{k}n\times 2^{k}n$ (k∈N). We added downblocks consisting of downsampling layers at the end of generator $G_{2}$ to scale down the feature maps, as shown in Fig. 3(b).

#### 2.3.2 Multi-modality super-resolution loss in SR-CycleGAN

There are two important factors in the SR of clinical CT images. One is anatomical structure, and the other is intensity distribution. Here, we explain the relationship between anatomical structure and intensity distribution. Structures such as arteries, bronchi, and alveoli are anatomical structures. Intensity distribution describes how a certain tissue has a certain intensity (grayscale). The intensity of clinical CT is described by the Hounsfield scale, and a specific substance such as bone has a specific intensity of +300 to +1900.^37^ On the other hand, the intensity of μCT changes with every scan, so the intensity of a specific substance varies slightly at each time of scan.

**Fig. 4:**
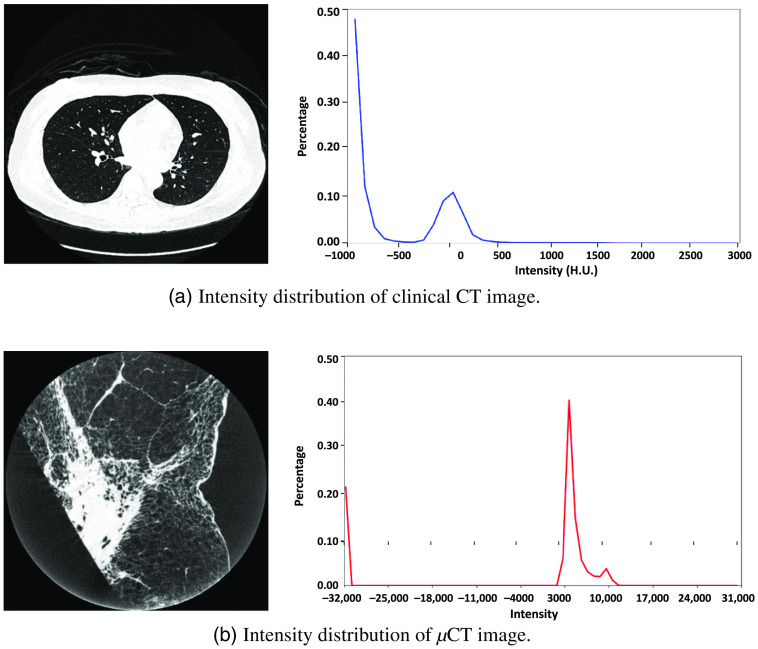
The intensity distribution of clinical CT image and μCT image. Intensity of clinical CT is described by the Hounsfield scale, and a specific substance such as bone has a specific intensity of +300∼+1900.^37^ The intensity of μCT is not described by the Hounsfield scale, and a specific substance’s intensity varies slightly at each time of scan. An example of a clinical CT image and its intensity distribution is shown in (a). An example of a μCT image and its intensity distribution is shown in (b). Histogram at right side: x axis is the intensity value of a particular pixel, while y-axis is the percentage of corresponding intensity. For the blue curve of the graph (a), around 0 H.U. on the x axis, the y axis is around 0.11. This implies that the number of voxels with an intensity of −100∼0 H.U. of clinical CT is around 11% of the total number of voxels. It is noteworthy that for clinical CT, we count the number of voxels by every one hundred, but since the intensity range of μCT is huge, we count the number of voxels here by every one thousand. The histograms illustrate that the intensity distributions of clinical and μCT are very different, which is one reason why CycleGAN without the proposed MMSR loss failed to perform SR of clinical CT using μCT images.

The same anatomical structures have totally different intensity distributions between clinical CT and μCT. For instance, in clinical CT images, the intensities of blood vessels and bronchus walls are around 0 and −500 Hounsfield units (H.U.). In μCT images, the intensities of blood vessels and bronchus walls are around 15,000 and 11,000 in the scanner used in our experiments. The intensity distribution of μCT focuses on a range of about [2000, 15,000] as shown in Fig 4(b), while the intensity of a lung’s clinical CT is distributed relatively uniformly in the range [−1000,500] as shown in Fig. 4(a). Even if we normalize the intensities of both μCT and clinical CT to the range [−1,1], the histograms of the two intensity distributions are still very different.

For the SR of medical images, a drastic change in image appearances may mislead clinicians. We need anatomical structures such as blood vessels and bronchi in clinical CT images (LR image) to maintain their original size and shape after SR. In addition, we have to ensure that the intensity distribution of the clinical CT’s SR result stays close to that of the original clinical CT image.

The loss function used in conventional CycleGAN does not ensure that input LR and output SR images have the same anatomical structures and intensity distribution. If we only modify the network structure of CycleGAN as shown in Sec. 2.3.1, the modified network outputs SR images with totally different intensity and anatomical structures from the input LR image. The objective of conventional CycleGAN is to output images close to the target domain instead of the source domain. In clinical CT image SR, the source domain is the LR domain (clinical CT) and the target domain is the SR domain (μCT). Therefore, CycleGAN with conventional loss terms outputs SR images with no similarity to the input LR image. Loss terms that guarantee that the output SR image has the same anatomical structures and intensity distribution as the input LR image are desired.

**Fig. 5:**
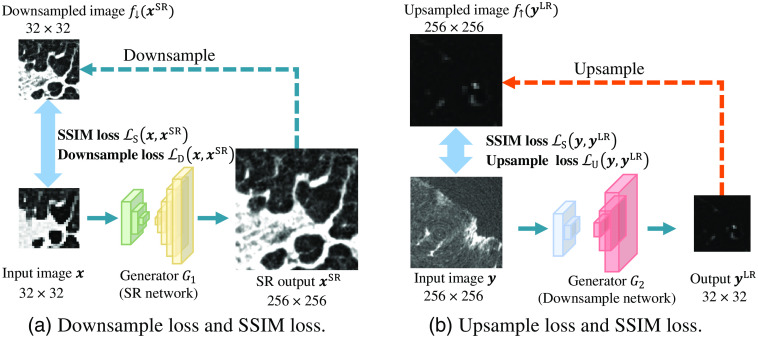
Illustration of proposed loss terms. SSIM loss and downsample loss between input clinical CT image x and output SR image $x^{\mathrm{SR}}$ are shown in (a). We use the average pooling function $f_{↓}()$ to downsample $x^{\mathrm{SR}}$ to the same size of input x. Then we calculate the SSIM loss and downsample loss of x and $f_{↓}(x^{\mathrm{SR}})$. These losses are calculated to optimize the parameters in generator $G_{1}$. SSIM loss and upsample loss between input μCT image y and output downsample image $y^{\mathrm{LR}}$ are shown in (b). We use the nearest upsampling function $f_{↑}()$ to upsample the generated clinical CT-like low-resolution image $y^{\mathrm{LR}}$. Then we calculate the SSIM loss and downsample loss of y and $f_{↑}(y^{\mathrm{LR}})$. These losses are calculated to optimize the parameters in generator $G_{2}$.

We propose a novel loss function named MMSR loss as shown in Fig. 5. The MMSR loss contains the following terms: (1) structural similarity (SSIM) loss, (2) downsample loss, and (3) upsample loss. As shown in Fig. 5, the downsample loss and upsample loss ensure that the SR image has a similar intensity distribution to that of the input LR image, and the SSIM loss ensures that the SR image has similar anatomical structures to those of the input LR image. Consequently, we use the MMSR loss to train SR-CycleGAN.

##### SSIM loss

The first loss term we propose is named SSIM loss. SSIM^38^ is an indicator that evaluates the structure similarity of two images. SSIM between two images is defined as

$$\mathrm{SSIM}(a,b)=\frac{\left(\mu_{a}\mu_{b}+C_{1})(2\sigma_{ab}+C_{2}\right)}{\left(\mu_{a}^{2}+\mu_{b}^{2}+C_{1})(\sigma_{a}^{2}+\sigma_{b}^{2}+C_{2}\right)},$$ (3)

where $\mu_{a}$ and $\mu_{b}$ are the average intensity of given images a and b, respectively. $\sigma_{a}$ and $\sigma_{b}$ are the variance of given images a and b, respectively. $\sigma_{\mathrm{ab}}$ is the covariance of given images a and b. $C_{1}$ and $C_{2}$ are constant numbers included to avoid instability. Based on this equation, we set the loss term named SSIM loss as

$$L_{S}(x,x^{\mathrm{SR}})=E_{x∼X}[1-\mathrm{SSIM}(x,f_{↓}(x^{\mathrm{SR}}))],$$ (4)

where x is an input clinical CT image, $x^{\mathrm{SR}}$ is the SR image, X is the domain of clinical CT images, and $f_{↓}()$ is the average pooling^39^ function. Average pooling calculates the average value for patches of a feature map and uses it to create a downsampled (pooled) feature map.^40^ $f_{↓}()$ rescales a given image to 1/n (n∈R) of its original size by width and height. We use $1-\mathrm{SSIM}(x,f_{↓}(x^{\mathrm{SR}}))$ as the basis of this loss term, since we desire the SSIM of x and $f_{↓}(x^{\mathrm{SR}})$ to be close to 1.

##### Downsample loss

To prevent a change of intensity in the CT image after SR, we propose another loss term named the downsample loss, which is written as

$$L_{D}(x,x^{\mathrm{SR}})=E_{x∼X}{\left\|(x,f_{↓}(x^{\mathrm{SR}}))\right\|}_{2}^{2},$$ (5)

where ${\left\|\cdot \right\|}_{2}^{2}$ is the square of the $l^{2}$-norm, x is the input clinical CT (LR) image, and $x^{\mathrm{SR}}$ is the SR image. We call this the downsample loss because it is calculated using the downsampled SR image $f_{↓}(x^{\mathrm{SR}})$ and the input LR image x. Since the downsample loss calculates the pixel-wise loss between the SR and LR images, this loss can prevent the SR image $x^{\mathrm{SR}}$ from deforming and changing of its intensity in relation to the LR image.

##### Upsample loss

The third proposed loss term is named upsample loss. As shown in Fig. 5(b), in SR-CycleGAN, there is another generator $G_{2}$ that can translate a given μCT image y into a clinical CT-like image $y^{\mathrm{LR}}$. By the same principle as downsample loss, to prevent a change in the intensity between y and $y^{\mathrm{LR}}$, the upsample loss is formulated as

$$L_{U}(y,y^{\mathrm{LR}})=E_{y∼Y}{\left\|(y,f_{↑}(y^{\mathrm{LR}}))\right\|}_{2}^{2},$$ (6)

where $f_{↑}()$ is the nearest upsampling function. The nearest upsampling function selects the value of the nearest pixels of a feature map, and then assigns this value to new pixels to create an upsampled feature map. $f_{↑}()$ rescales a given image to k (k∈R) times its original size by width and height, and Y is the domain of μCT images y. We call this the upsample loss because it is calculated from the $l^{2}$ norm between the upsampled fake clinical CT $f_{↑}(y^{\mathrm{LR}})$ and the original μCT y.

##### Adding MMSR loss in SR-CycleGAN

The MMSR loss consists of SSIM loss, downsample loss, and upsample loss. The MMSR loss is formulated as

$$L_{\mathrm{MMSR}}(x,y,y^{\mathrm{LR}},x^{\mathrm{SR}})=\lambda_{1}L_{S}(x,f_{↓}x^{\mathrm{SR}}))\;+\lambda_{2}L_{S}(y,f_{↑}(y^{\mathrm{LR}}))\;+\lambda_{3}L_{D}(x,f_{↓}(x^{\mathrm{SR}}))\;+\lambda_{4}L_{U}(y,f_{↑}(y^{\mathrm{LR}})),$$ (7)

where $L_{S}(x,f_{↓}(x^{\mathrm{SR}}))$ is the SSIM loss between the input clinical image x and the output SR image $x^{\mathrm{SR}}$. $L_{S}(y,f_{↑}(y^{\mathrm{LR}}))$ is the SSIM loss between the μCT image y and the generated clinical CT-like image $y^{\mathrm{LR}}$. $L_{D}(x,f_{↓}(x^{\mathrm{SR}}))$ is the downsample loss of x and $x^{\mathrm{SR}}$. $L_{U}(y,f_{↑}(y^{\mathrm{LR}}))$ is the upsample loss of y and $y^{\mathrm{LR}}$. $f_{↓}()$ is the average pooling function that scales up a given image. $f_{↑}()$ is the nearest upsampling function that scales down a given image. $\lambda_{1}$, $\lambda_{2}$, $\lambda_{3}$, and $\lambda_{4}$ are weights. We add the proposed MMSR loss as an additional loss term into the proposed SR-CycleGAN. We formulate the total loss function of SR-CycleGAN as

$$L_{\text{Total}}=\lambda_{1}L_{S}(x,f_{↓}(x^{\mathrm{SR}}))\;+\lambda_{2}L_{S}(y,f_{↑}(y^{\mathrm{LR}}))\;+\lambda_{3}L_{D}(x,f_{↓}(x^{\mathrm{SR}}))\;+\lambda_{4}L_{U}(y,f_{↑}(y^{\mathrm{LR}}))\;+\lambda_{5}L_{\mathrm{GAN}}(x^{\mathrm{SR}},y)\;+\lambda_{6}L_{\mathrm{GAN}}(y^{\mathrm{LR}},x)\;+\lambda_{7}L_{\mathrm{cyc}}(x,G_{2}(x^{\mathrm{SR}}),y,G_{1}(y^{\mathrm{LR}})),$$ (8)

where $L_{\mathrm{GAN}}(x^{\mathrm{SR}},y)$ and $L_{\mathrm{GAN}}(y^{\mathrm{LR}},x)$ are GAN loss, and $L_{\mathrm{cyc}}(x,G_{2}(x^{\mathrm{SR}}),y,G_{1}(y^{\mathrm{LR}}))$ is cycle-consistency loss proposed in the conventional CycleGAN described in Sec. 2.2. $\lambda_{5}$, $\lambda_{6}$, and $\lambda_{7}$ are weights. By adding the MMSR loss to CycleGAN, we successfully performed the SR of clinical CT of lung cancer patients to the μCT level, while conventional CycleGAN failed to perform SR.

### 2.4 Training and Inference of SR-CycleGAN

In the training phase, the input of generator $G_{1}$ is a clinical CT image with the size of n×n pixels. We denote the clinical CT image as x. The generator $G_{1}$ generates an SR image $x^{\mathrm{SR}}=G_{1}(x)$ with a size of $2^{k}n\times 2^{k}n$ pixels. On the other hand, a μCT image y with a size of $2^{k}n\times 2^{k}n$ pixels is input into the generator $G_{2}$. The generator $G_{2}$ generates a clinical CT-like image $y^{\mathrm{LR}}=G_{2}(y)$ of n×n pixels from the μCT image y of $2^{k}n\times 2^{k}n$ pixels. The loss of the entire SR-CycleGAN is calculated from x, $x^{\mathrm{SR}}$, y, and $y^{\mathrm{LR}}$. Then the loss is used for to optimize the network.

For inference, we only use the trained generator $G_{1}$. We extracted images of size n×n pixels from clinical CT and input them into the trained network $G_{1}$. The output is SR images of size $2^{k}n\times 2^{k}n$ pixels.

## 3 Experiments and Results

### 3.1 Datasets

**Fig. 6:**
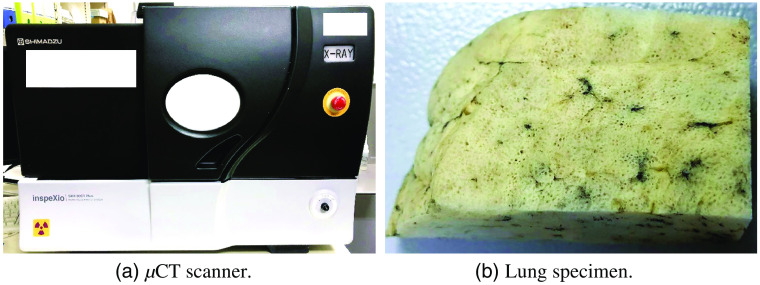
Our μCT scanner and a sample of lung specimen. μCT scanner (inspeXio SMX-90 CT Plus, Shimadzu Inc., Kyoto, Japan) is shown in (a). Resected lung cancer specimen from human lung cancer patient is shown in (b).

In our experiments, we newly built a dataset containing ten μCT volumes and eight clinical CT volumes. The clinical CT volumes were scanned by a clinical CT scanner (SOMATOM Definition Flash, Siemens Inc., Munich, Germany). The resolution of the clinical CT volumes was $0.625\times 0.625\times 0.6\;{\mathrm{mm}}^{3}/\text{voxel}$. The size of the clinical CT volumes was 512×512×435∼554  voxels. The μCT volumes were scanned by a μCT scanner (inspeXio SMX-90 CT Plus, Shimadzu Inc., Kyoto, Japan) as shown in Fig. 6(a). The lung cancer specimens were fixed by Heitzman’s method^41^ as shown in Fig. 6(b). Lung specimens were scanned at isotropic resolutions of $42∼52\times 42∼52\times 42∼52\;\mu m^{3}/\text{voxel}$. The size of the μCT volumes was 1024×1024×545∼983  voxels. We trained SR-CycleGAN using five clinical CT volumes and five corresponding μCT volumes of lung cancer specimens. We evaluated the SR-CycleGAN qualitatively on three clinical CT volumes and quantitatively on five μCT volumes. These clinical and μCT volumes were not used for training.

### 3.2 Preprocessing

Chest clinical CT images have various tissues outside the lungs that are not appropriate for our experiments, such as bones, muscles, esophagus, etc. We first segmented lung regions from clinical CT chest images. We conducted region growing^42^ to obtain a coarse segmentation mask of the lung and performed morphological operations to fill the holes in the coarse segmentation mask.

μCT images also require a target region restriction. In our experiments, lung specimens were placed in a plastic cylinder and put into the μCT scanner for scanning. Therefore, parts of the plastic cylinder are shown in the μCT images. Since the plastic cylinder is not suitable for our experiment, we manually cropped lung regions from the μCT images, and only used the lung regions for the experiment.

In addition, normalization of the intensities of both clinical CT and μCT images was required. We normalized both the intensity of μCT and clinical CT to the range [−1,1]. In clinical CT, the intensity of a tissue is represented using the Hounsfield scale, with water having a value of 0 H.U., tissues denser than water having positive values, and tissues less dense than water having negative values.^43^ In μCT, the intensity is not represented by Hounsfield scale. The intensity range of the clinical CT volume was about 3500 H.U. (intensity of air is around −1000 H.U. and intensity of bone is around 2500 H.U.), but the scale of the μCT volume was about 16,000 (intensity of air is around −1000 to 0, and cancer is around 15,000). For clinical CT, we normalized the intensity in this way: For intensity larger than 2500 H.U. (larger than the bone intensity), we set the intensity to 2500 H.U. We also set voxels that have intensity smaller than −1000 H.U. to −1000 H.U. For μCT, we set voxels that have intensity higher than 15,000 (higher than cancer) to 15,000 and set voxels that have intensity smaller than 0 to 0. Finally, the intensities of both clinical CT and μCT images were compressed to [−1,1].

### 3.3 Parameter Settings

#### 3.3.1 SR rate and training patch numbers

Conventionally, SR was conducted $2^{k}$ (k∈N) times, which means the SR image was $2^{k}$ (k∈N) times larger than the LR image. Considering the resolution of clinical CT volumes (625 mm) and μCT volumes (52 mm), we chose 8× SR. In the training phase, we extracted 2000 patches with a size of 32×32  pixels randomly from each clinical CT case. We also extracted 2000 patches of the size of 256×256  pixels randomly from each μCT case. Since we had five cases for training, the total numbers of clinical and μCT patches were both 10,000.

#### 3.3.2 Parameters for network training

We used Adam^44^ for stochastic optimization of the network. We set the learning rate to $10^{-5}$, while the training rate remained $10^{-5}$ from 1 to 100 epochs, and decayed linearly from $10^{-5}$ to 0 between 100 to 200 epochs. The mini-batch size of training was 4. Training was continued until 200 epochs. We manually chose weights λ of each loss term that could obtain the best qualitative results on the training dataset. Weights λ of each loss term are listed in Table 1. All networks were implemented by PyTorch.

**Table 1:** Parameters of each loss term.

$\lambda_{1}$: weight for SSIM loss of $G_{1}$	1.0
$\lambda_{2}$: weight for SSIM loss of $G_{2}$	1.0
$\lambda_{3}$: weight for downsample loss	0.7
$\lambda_{4}$: weight for upsample loss $G_{1}$	0.3
$\lambda_{5}$: weight for GAN loss of $G_{1}$ and $D_{1}$	1.0
$\lambda_{6}$: weight for GAN loss of $G_{2}$ and $D_{2}$	1.0
$\lambda_{7}$: weight for cycle-consistency loss	1.0

#### 3.3.3 Evaluation methods

For qualitative evaluation, we utilized three clinical CT volumes. We cropped clinical CT images of size 32×32  pixels from three clinical CT volumes and input the clinical CT images into generator $G_{1}$ of trained SR-CycleGAN. Then, we obtained SR images of size 256×256  pixels. For demonstrating the effectiveness of network modification and MMSR loss of SR-CycleGAN, we compared SR-CycleGAN with conventional CycleGAN. Since input and output of CycleGAN is of the same size, CycleGAN could not be applied directly for SR. Therefore, we add upblocks into CycleGAN’s generator $G_{1}$ to ensure output of $G_{1}$ is eight times larger than input (by width and height). We name this CycleGAN as “CycleGAN with upblocks.” We also conducted ablation experiments to verify the effectiveness of network modification.

For quantitative evaluation, we proposed a novel quantitative evaluation method. In previous supervised SR studies,^45^ quantitative evaluations were often conducted by comparing the output SR image with its HR counterpart. Therefore, paired LR images (clinical CT images) and HR images (μCT images) were required for quantitative evaluations. Since we could not obtain paired clinical CT/μCT images, we conducted an alternative approach: First, we used bicubic interpolation^31^ to downsample μCT images to 1/8 of their original size to simulate clinical CT images (In image processing, bicubic interpolation is used for interpolating data points on a 2D regular grid. Bicubic interpolation considers 16 pixels (4×4) around the pixel to be interpolated and calculates a weighted addition of these 16 pixels as the new pixel.). For a given μCT image of 256×256  pixels, we performed bicubic downsampling of the μCT image to obtain an image size of 32×32  pixels and then input it into trained $G_{1}$ to obtain a 256×256  pixel SR output. We compared the SR output with the original μCT images using evaluation metrics such as peak signal-noise ratio (PSNR).^46^ It is noteworthy that $G_{1}$ is trained by clinical CT and μCT images as explained in Sec. 3.3.1. We used five μCT cases of 1544 images for quantitative evaluation.

We compared the following networks. Network1: CycleGAN with upblocks (no MMSR loss, no network modification, only upblocks for a larger output image). Network2: CycleGAN with network modification (sub-pixel shuffling layers but no MMSR loss). Network3: SR-CycleGAN with downblocks (with MMSR loss, no sub-pixel shuffling layers). Network4: Proposed SR-CycleGAN (with MMSR loss and sub-pixel shuffling).

**Fig. 7:**
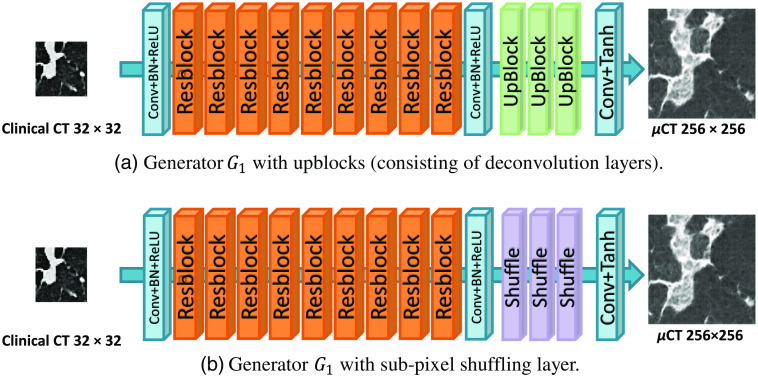
To prove that the sub-pixel shuffling layers actually reduce computing time, we performed experiments on two kinds of generator $G_{1}$: (a) generator $G_{1}$ with upblocks and (b) generator $G_{1}$ with sub-pixel shuffling layers. We extracted 2000 patches for training on Nividia Tesla V100 (32 GB memory). (a) needed 491 s for training in each epoch, while (b) needed 353 s in each epoch.

We also quantitatively evaluated how sub-pixel shuffling layers reduce training time. Before adding sub-pixel shuffling layers in generator $G_{1}$, we used upblocks to upscale the feature maps to a larger size. Figure 7 shows a comparison of $G_{1}$ with/without pixel-shuffling layers. We used 2000 patches cropped from clinical CT images of 32×32  pixels and 2000 patches cropped from μCT images of SR-CycleGAN for training.

### 3.4 Comparison of Results

**Fig. 8:**
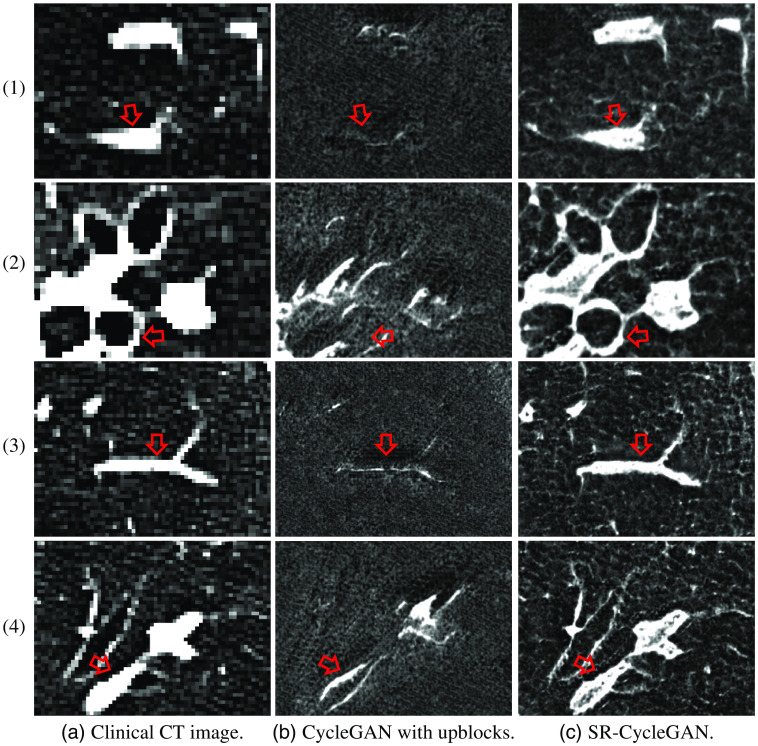
SR results of clinical CT images from one case. Rows (1) and (3) are images cropped from blood vessels and lung field region. Rows (2) and (4) are images cropped from the bronchus and blood vessels region. Column (a) are original clinical CT images. Column (b) and (c) are results of “CycleGAN with upblocks” and our method, respectively. We can obtain that SR-CycleGAN output reliable SR results, while CycleGAN with upblocks (no MMSR loss, no network modification, only upblocks for larger output image) output results that do not have similarity with the input image. As pointed by red arrows, blood vessels and bronchus in SR images of CycleGAN with upblocks severely deformed or disappeared, while blood vessels and bronchus in SR-CycleGAN’s SR images have sharp edges and same shape as in LR images.

**Fig. 9:**
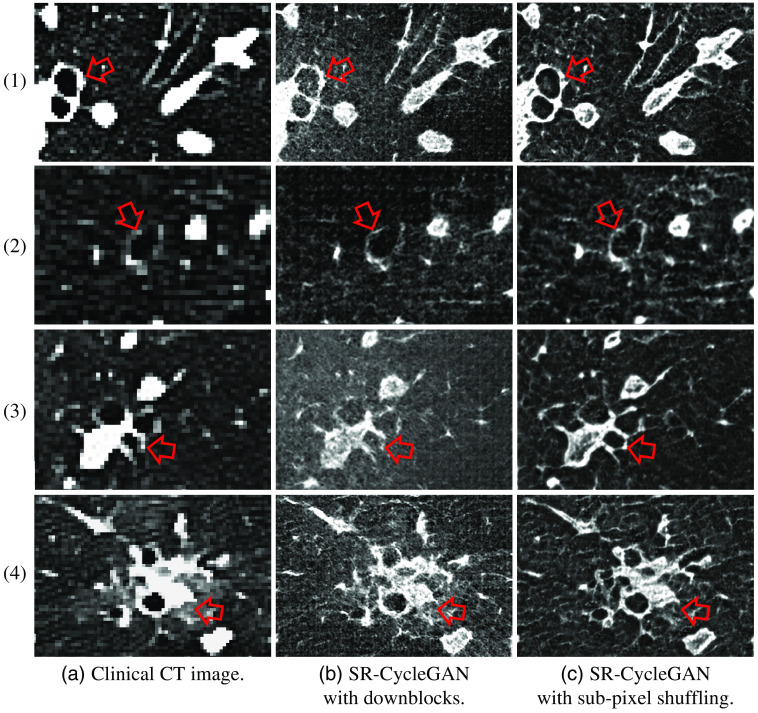
Comparison of SR-CycleGAN before/after removing downblocks and adding sub-pixel shuffling layers. Rows (1), (2), and (3) are CT images of the bronchus and blood vessel region. Row (4) has CT images of the tumor and bronchus region. Column (a) are clinical CT images. Column (b) and (c) are results of “SR-CycleGAN with downblocks” and “SR-CycleGAN with sub-pixel shuffling” respectively. After removing downblocks and adding sub-pixel shuffling layers, SR-CycleGAN performed better qualitatively. As indicated by the red arrows, results of SR-CycleGAN with downblocks (SR-CycleGAN with downblocks and before adding sub-pixel shuffling layers) have many artifacts, and the edges of the bronchus and blood vessels look discontinuous. On the other hand, these defects do not appear in the results of SR-CycleGAN.

SR results of SR-CycleGAN were compared with CycleGAN with upblocks in Fig. 8. Furthermore, for evaluating the effectiveness of removing downblocks and introducing sub-pixel shuffling layers, we also evaluated SR-CycleGAN with/without removing downblocks and with/without sub-pixel shuffling layers as shown in Fig. 9.

#### 3.4.1 Qualitative evaluation

We show the cropped part of the SR images obtained by the SR-CycleGAN in Fig. 8(c). The results of CycleGAN with upblocks are shown in Fig. 8(b). In SR results of SR-CycleGAN, lung anatomies, such as the bronchus, appear more clearly than the original clinical CT images as indicated by red arrows in Fig. 8(c). CycleGAN with upblocks (no network modification except adding upblocks and no MMSR loss) only produced results that have no similarity with the input LR image (clinical CT image). Important anatomical structures such as the blood vessels and bronchus disappeared, as indicated by red arrows in Fig. 8(b). The results demonstrate that the proposed SR-CycleGAN is suitable for SR of clinical CT images.

The results of “SR-CycleGAN with downblocks”^47^ (SR-CycleGAN with MMSR loss but without network modification) are shown in Fig. 9(b), which seems noisy, and the edge of the blood vessel and bronchus has many artifacts indicated by red arrows. The results of SR-CycleGAN are shown in Fig. 9(c), which is clearer and noiseless compared with Fig. 9(b).

**Fig. 10:**
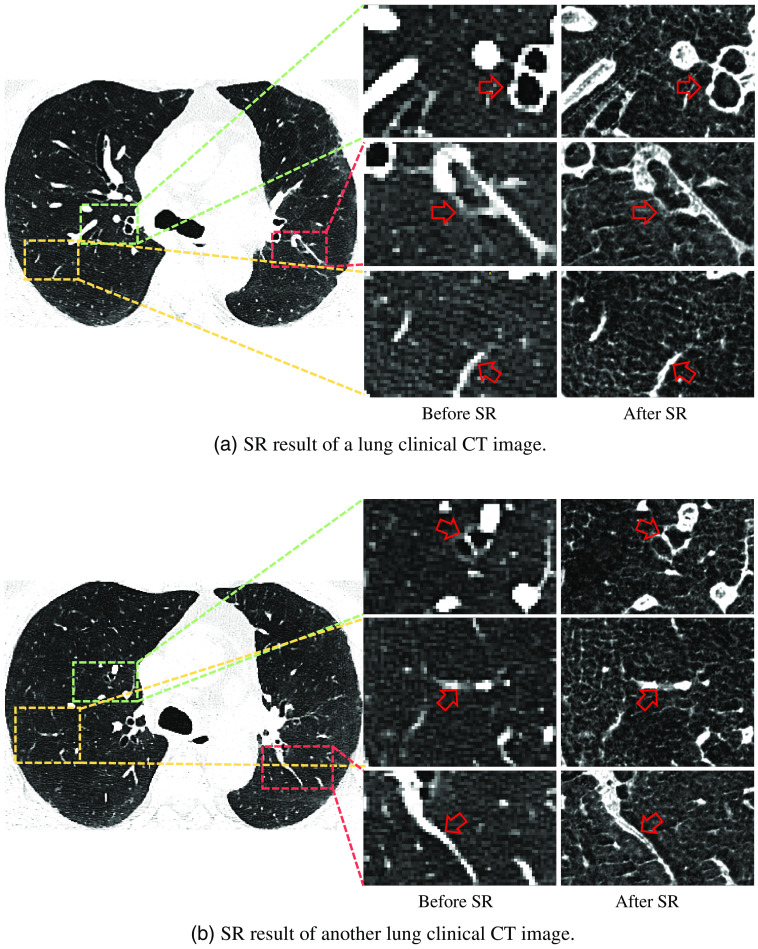
To observe SR results from larger scale, this image illustrates both clinical CT images of whole lung region and images cropped from lung region before and after SR. (a) CT image extracted from axial axis. (b) Another CT image extracted from axial axis. In (a) and (b), edges of arteries and bronchus (red arrows) are smoother and clearer after SR.

To observe SR results from a larger scale, we illustrate both clinical CT images of the whole lung region and images cropped from the lung region before and after SR in Fig. 10.

#### 3.4.2 Quantitative evaluation

**Fig. 11:**
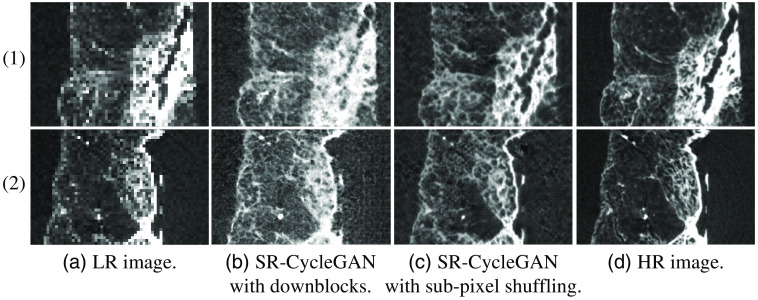
Qualitative results of SR-CycleGAN. SR results of SR-CycleGAN with downblocks are shown in (b), with PSNR of 16.48 dB. SR results of SR-CycleGAN with sub-pixel shuffling layer and without downblocks are shown in (c), with PSNR of 17.71 dB. Column (a) and (d) are LR images and corresponding HR images, respectively. We used bicubic downsampling^31^ to rescale μCT images (HR image) to 1/8 of their original sizes to simulate clinical CT images (LR images), and then input the downsampled image into trained SR-CycleGAN’s generator $G_{1}$. It is noteworthy that the higher PSNR indicates a better result.

The SR results and quantitative evaluation results are shown in Fig. 11 and Table 2. We used PSNR and SSIM^46^ for quantitative evaluation. Table 2 shows that the proposed SR-CycleGAN performed quantitatively better than other methods, with the highest PSNR and SSIM.

**Table 2:** Quantitative evaluation of our methods. Network1: CycleGAN with upblocks (no MMSR loss, no network modification, only upblocks for larger output image). Network2: CycleGAN with network modification (sub-pixel shuffling layers but no MMSR loss). Network3: SR-CycleGAN with downblocks (with MMSR loss, no sub-pixel shuffling layers). Network4: proposed SR-CycleGAN (with MMSR loss and sub-pixel shuffling). Bold values are the highest.

	Network1	Network2	Network3	SR-CycleGAN (our method)
PSNR	13.64	15.39	16.48	**17.71**
SSIM	0.05	0.37	0.44	**0.54**

We also evaluated how sub-pixel shuffling layers reduce training time. SR-CycleGAN without sub-pixel shuffling layers needs 491 s for training per epoch (2000 patches per epoch). After replacing upblocks with sub-pixel shuffling layers, the entire network needs 353 s for training per epoch. Thus, training time was significantly reduced. The network was trained on Nvidia Tesla V100 (32 GB memory).

### 3.5 Ablation Studies

For accessing the effectiveness of different components of our method, we performed ablation studies. On top of baseline (CycleGAN with upblocks), we progressively added network modification and the MMSR loss function. Further, to clear effectiveness of each component of MMSR loss, we also analyzed each term in MMSR loss separately. Experiments showed that our method with all proposed components performed best quantitatively and qualitatively.

#### 3.5.1 Effectiveness of network modification

**Fig. 12:**
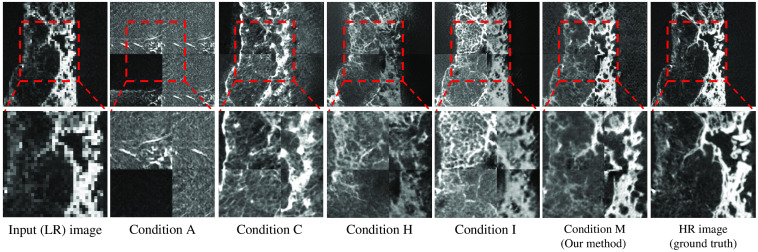
Qualitative results of ablation studies. We chose five combinations of each proposed component and illustrate the qualitative results of each combination in this figure. The method with all components (our method) achieved the highest PSNR and SSIM score. A, C, H, I, and M (our method) correspond to the “condition” column of Table 3. Upper: whole images. Lower: zoom-in on the regions in the red boxes for better comparison.

We first analyzed the effect of network modification. As network modification, we removed downblocks and added pixel-shuffling layers to a conventional CycleGAN’s generator $G_{1}$. Network modification avoided the need to encode the input image into smaller feature maps, thus preserving spatial information while performing SR. Additionally, it also reduced training and referencing time. With network modification, PSNR increased by 1.75 dB and SSIM increased by 0.32 compared to the baseline (CycleGAN with upblocks). The qualitative results of baseline and baseline with network modification are shown as condition A and condition C, respectively, in Fig. 12; images of the latter were qualitatively better than those of the former. Quantitative results of network modification are shown in Table 3. In Table 3, the PSNR and SSIM score of condition C (baseline with network modification) are higher than those of condition A (baseline). Therefore, network modification is required in our method.

**Table 3:** Ablation studies and quantitative results. SSIM loss 1 is $L_{S}(x,f_{↓}(x^{\mathrm{SR}}))$ and SSIM loss 2 is $L_{S}(y,f_{↑}(y^{\mathrm{LR}}))$. Applying network modification and all loss terms simultaneously obtains the highest PSNR and SSIM means such a component is not utilized, and F0FC means such a component is utilized. Bold values are the highest.

Condition	Network modification	Upsample loss	Downsample loss	SSIM loss 1	SSIM loss 2	PSNR	SSIM
A	—	—	—	—	—	13.64	0.05
B	—	✓	✓	✓	✓	16.48	0.44
C	✓	—	—	—	—	15.39	0.37
D	✓	✓	—	—	—	15.83	0.40
E	✓	—	✓	—	—	16.53	0.42
F	✓	—	—	✓	—	15.13	0.27
G	✓	—	—	—	✓	14.18	0.25
H	✓	✓	✓	—	—	15.92	0.40
I	✓	—	—	✓	✓	16.92	0.49
J	—	✓	✓	✓	✓	16.48	0.44
K	✓	—	✓	✓	✓	15.78	0.46
L	✓	✓	—	✓	✓	17.70	0.50
M (our method)	✓	✓	✓	✓	✓	**17.71**	**0.54**

#### 3.5.2 Effectiveness of MMSR loss

We analyzed the effectiveness of the proposed MMSR loss. The MMSR loss ensures that the output SR image has similar pixel-wise intensity distribution to that of the input LR image. The MMSR loss also prevents the network from generating arbitrary outputs. With the MMSR loss, PSNR increased by 2.84 dB; SSIM increased by 0.39 compared to the method without MMSR loss.

We further studied the effectiveness of each loss term in the MMSR loss. The MMSR loss contains the following components: (1) SSIM loss (containing two loss terms), (2) downsample loss, and (3) upsample loss. Upsample loss and downsample loss ensure that the output SR image has a higher pixel-wise similarity with the input image. SSIM loss ensures that the output image has a higher structural similarity^48^ with the input image. We studied various combinations of loss terms and show their quantitative results in Table 3. In Table 3, each loss term in MMSR loss brought an increase in PSNR and SSIM, and the SSIM loss (containing two loss terms) brought more improvement than other loss terms (condition I in Table 3). We chose four combinations of loss terms (conditions A, H, I, and M in Table 3) whose qualitative results have huge differences. The qualitative evaluation results of these four combinations are shown in Fig. 12, which shows that our method’s output (condition M) has the highest similarity with the HR image (ground truth), compared with the other combinations of loss terms (conditions A, C, H, and I).

### 3.6 Comparison with Recent Baselines

**Fig. 13:**
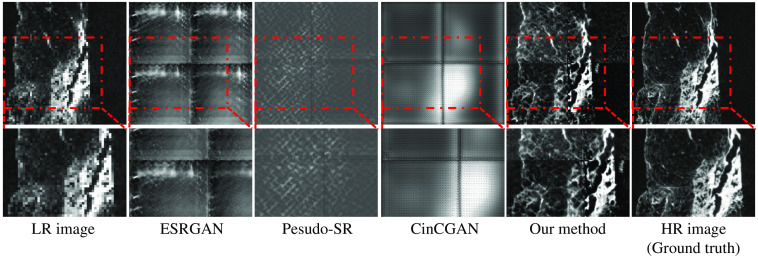
Qualitative comparison between our method and recent baselines on clinical CT−μCT dataset. We compared our method with a recent supervised baseline (ESRGAN^51^) and two recent unsupervised baselines (pseudo-SR^49^ and CinCGAN).^32^ Our method output convincing SR results, while recent SR baselines output SR images quite different from the HR images (ground truth). Upper: whole images. Lower: zoom-in on regions in the red boxes for better comparison.

We compared our method with three recent SR methods. We first compared our method with a recent unsupervised baseline named CinCGAN.^32^ CinCGAN first utilizes cycle-in-cycle network structure to map a noisy and blurry LR image to a noise-free LR image. Then the noise-free LR image is upsampled with a pre-trained deep SR model. CinCGAN is trained with LR-HR images in an end-to-end manner. The trained CinCGAN is used for performing SR of a given LR image.^32^ We also compared our method with a newly proposed SOTA unsupervised SR method named pseudo-SR,^49^ and a widely used supervised SR method named ESRGAN.^23^ Pseudo-SR is an SR method consists of an unpaired kernel/noise correction network and a pseudo-paired SR network. The correction network removes noise and adjusts the blurring kernel^50^ of the input LR image. Then the pseudo-paired SR network upscales the corrected clean LR image.^49^ ESRGAN is a supervised SR method utilizing newly proposed loss terms such as adversarial loss and perceptual loss, and the residual-in-residual dense block into SR network.^51^ We did not have paired clinical CT (LR) and μCT (HR) images. Therefore, we trained ESRGAN with unpaired LR-HR images. The results of our method and these recent baselines were shown in Fig. 13. As shown in the red boxes in Fig. 13, our method output SR images close to the HR images (ground truth). Recent SR baselines output SR images quite different from the HR images (ground truth). The PSNR and SSIM of our method were the highest among all methods, as shown in Table 4. We also compared our method’s inference time, training time, and parameter size with recent baselines in Table 5. As shown in the Table 5, training time for one epoch was the shortest with our method, and the number of network parameters was the smallest.

**Table 4:** Quantitative comparison between our method and recent baselines. Our method has the highest PSNR and SSIM score. These results were computed on the clinical CT−μCT dataset. Bold values are the highest.

	ESRGAN^51^	Pseudo-SR^49^	CinCGAN^32^	Our method
PSNR	15.32	11.08	9.99	**17.71**
SSIM	0.02	0.04	0.31	**0.54**

**Table 5:** Comparison of training time, inference time and number of parameters between our method and recent baselines. Our method has the shortest average training time and the fewest parameters compared to recent SR baselines ESRGAN,^51^ pseudo-SR,^49^ and CinCGAN.^32^ Bold values are the highest.

	ESRGAN^51^	Pseudo-SR^49^	CinCGAN^32^	Our method
Average training time (1 epoch)	3 h 41 min	9 h 47 min	12 h 13 min	**40 min**
Inference time	4 min 59 s	8 min 32 s	**3 min 41 s**	4 min 27 s
Number of network parameters	24,383,820	32,995,229	27,030,790	**19,264,369**

### 3.7 Experimental Results on COVID-19 Lung CT Segmentation Challenge—2020 Dataset

**Fig. 14:**
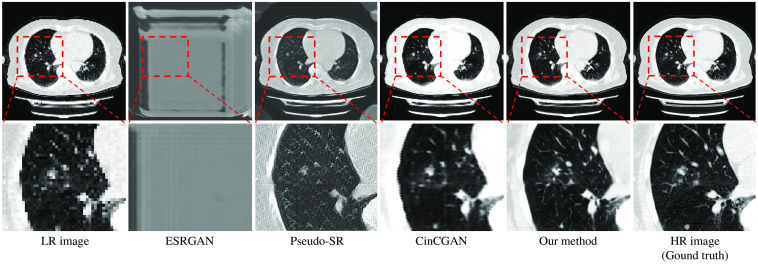
Experimental result on COVID-19 Lung CT Lesion Segmentation Challenge—2020 dataset.^52^ We compared our method with ESRGAN,^51^ pseudo-SR,^49^ and CinCGAN.^32^ It is noteworthy that because our method is trained with unpaired LR-HR images pairs, we also train ESRGAN with unpaired LR-HR images. ESRGAN output unreasonable results. Pseudo-SR and CinCGAN output blurry and noisy results. On the other hand, our method output convincing results. Upper: whole images from the axial axis. Lower: zoom-in on regions in the red boxes for better comparison.

We also performed an experiment with an additional benchmark CT dataset to examine whether our method could perform SR of commonly used medical images (such as CT images). We chose the COVID-19 Lung CT Segmentation Challenge—2020 dataset.^52^ This dataset has 249 cases collected from patients of different hospitals, countries, ages, and genders. Here, 199 cases were for training and 50 cases were for testing. We chose 4× SR (width and length of an output image are four-times those of an input image). Input LR image size was 48×48  pixels, and output SR image size was 192×192  pixels. We compared our method with recent baselines: unsupervised SR methods CinCGAN^32^ and pseudo-SR,^49^ and a supervised method ESRGAN.^51^ Qualitative results are shown in Fig. 14, and quantitative results are shown in Table 6. Our method outperformed these recent baselines quantitatively as shown in Table 6. It could output clear images and reconstruct important anatomical structures such as vessels and bronchi. Results of recent baselines are blurred (CinCGAN and pseudo-SR) or unreasonable (ESRGAN) in Fig. 14. The experimental results prove that our method is effective on commonly used medical images.

**Table 6:** Quantitative comparison between our method and recent baselines on the COVID-19 Lung CT Segmentation Challenge—2020 dataset.^52^ Bold values are the highest.

	ESRGAN^51^	Pseudo-SR^49^	CinCGAN^32^	Our method
PSNR	7.47	17.68	23.26	**26.10**
SSIM	0.22	0.88	0.97	**0.98**

## 4 Discussions

### 4.1 Unsupervised SR of Clinical CT Utilizing μCT Data

To the best of our knowledge, our method is the first method to perform SR on clinical CT to the μCT scale without a corresponding HR image as ground truth. The method is also the first to perform SR of clinical CT utilizing μCT data. MMSR loss and modification of networks enabled SR-CycleGAN to perform SR by forcing SR images to have the same anatomical structures as the input clinical CT (LR) images. We believe MMSR loss is more important than network modification, since in Fig. 8(b), CycleGAN with upblocks (no MMSR loss, no network modification, only upblocks for larger output image) output results that do not have similarity with the input images. As shown in Fig. 9(b), SR-CycleGAN with downblocks (with MMSR loss, no network modification) performed SR of clinical CT images. However, these results were not as good as SR-CycLeGAN with sub-pixel shuffling (with both MMSR loss and network modification) in Fig. 9(c). MMSR loss enabled SR of clinical CT images, and modification of the network enhanced the qualitative and quantitative results.

### 4.2 Effect of Hyperparameter Adjustment

**Fig. 15:**
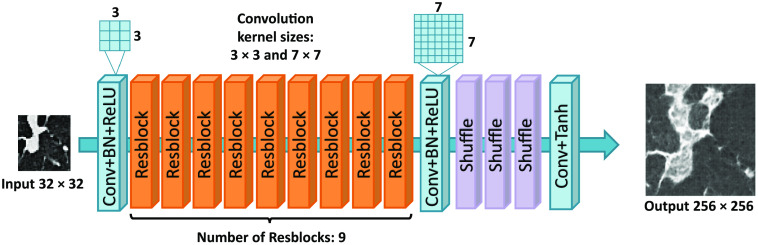
Hyperparameters of our method’s generator $G_{1}$. The first Conv+BN+ReLU block uses a convolution kernel of size 3×3 and the second Conv+BN+ReLU block uses a convolution kernel of size 7×7. Input patch size is 32×32  pixels and output size is 256×256  pixels. Number of Resblocks is 9.

We performed further experiments to address the effect of different hyperparameters on the final result. Specifically, we changed the number of Resblocks, the convolution kernel size, and the patch size for training. We showed the number of Resblocks, the convolution kernel size, and the patch size utilized in our method in Fig. 15. First, we changed the number of Resblocks. The number of Resblocks in generator $G_{1}$ of our method was 9. Since we built our method based on CycleGAN, whose numbers of Resblocks were 6 (for small patches) and 9 (for large patches), we performed an experiment with a smaller number of Resblocks 6. In addition, since the difference between 9 (number of Resblocks in our method) and 6 (the smaller number of Resblocks) was 3, we further performed an experiment with a larger number of Resblocks of 9+3=12. Furthermore, we performed an experiment with a larger or smaller convolution kernel. The first Conv+BN+ReLU block in generator $G_{1}$ of our method utilized a convolution kernel of size 3×3; the second Conv+BN+ReLU block utilized a convolution kernel of size 7×7. We changed the first Conv+BN+ReLU block’s convolutional kernel size to 7×7 to test the effect of a larger convolution kernel. Correspondingly, we changed the second Conv+BN+ReLU block’s convolutional kernel size to 3×3 to test the effect of a smaller convolution kernel. The patch size for training was also adjusted. The input patch size in our method was 32×32  pixels. We tried using smaller (24×24  pixels) and larger (48×48  pixels) patch sizes to investigate the impact of patch size on the results.

**Fig. 16:**
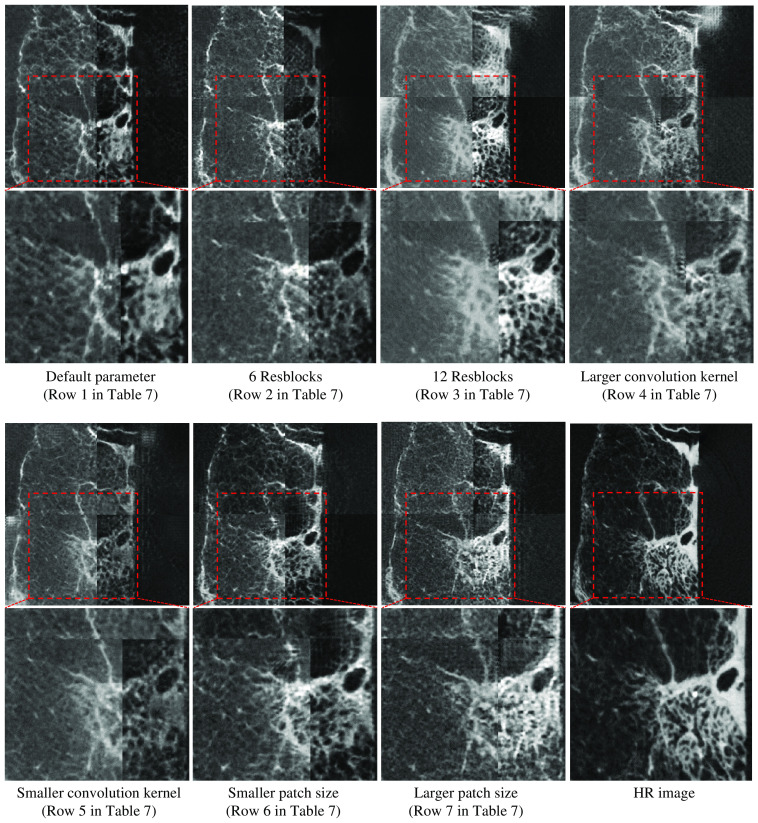
Results of different hyperparameter settings. We performed an experiment with generator $G_{1}$ with different numbers of Resblocks, different convolution kernel sizes, and different patch sizes. Table 7 gives detailed parameters. We zoom in on the regions in the red boxes for a better comparison.

Table 7 shows that using 9 Resblocks, 3×3 and 7×7 convolution kernel sizes, and 32×32  pixels patch size led to the highest PSNR and SSIM score. Using either more or fewer Resblocks, larger or smaller convolution kernel size, or larger or smaller patch size resulted in a lower PSNR and SSIM score. Qualitative results of different hyperparameters were similar, as shown in Fig. 16. It is obvious that the parts enclosed in the red boxes in Fig. 16 do not have significant differences. In conclusion, the experimental results show that our method’s number of Resblocks, convolution kernel sizes, and patch size resulted in the best quantitative result as shown in Table 7. Additionally, the number of Resblocks, convolution kernel sizes, and patch size do not have much effect on the qualitative results as shown in Fig. 16.

**Table 7:** Different hyperparameters result in different experimental results. Experimental results showed that using nine Resblocks, 3×3 and 7×7 convolution kernel sizes, and 32×32  pixels patch size results in the best PSNR and SSIM score. The red characters in each condition indicate its difference with condition 1. Bold values are the highest.

Condition	Number of Resblocks	Convolution kernel size	Patch size	PSNR	SSIM
1	9	3 × 3 and 7 × 7	32×32 pixels	**17.71**	**0.54**
2	6	3 × 3 and 7 × 7	32×32 pixels	17.49	0.44
3	12	3 × 3 and 7 × 7	32×32 pixels	15.36	0.45
4	9	7 × 7 and 7 × 7	32×32 pixels	16.73	0.41
5	9	3 × 3 and 3 × 3	32×32 pixels	15.20	0.43
6	9	3 × 3 and 7 × 7	24×24 pixels	16.04	0.36
7	9	3 × 3 and 7 × 7	48×48 pixels	17.52	0.53

### 4.3 Novelty of Our Method and Difference from Recent CT SR Methods

Our method has three novel components: (1) a lightweight network equipped with sub-pixel shuffling layers,^36^ (2) novel loss terms named upsample and downsample losses, and (3) a novel loss term named SSIM loss. We modified components (1), (2), and (3) in applying them to our task. We added component (1) in CycleGAN to apply component (1) in unsupervised scenarios. Although components (2) and (3) have been used as loss terms in some SR methods,^53^ they were never used to measure the similarities of different-size images (e.g., one image size of 32×32 and another of 128×128). We modified components (2) and (3) to measure the similarities of differently sized images and utilized the similarities as loss terms to optimize our proposed network. No existing CT SR method utilizes components (1), (2), and (3) at the same time. By combining components (1), (2), and (3) in our method, we successfully implemented unsupervised SR with a relatively lightweight network. As a result, our method successfully achieved SR on a clinical CT−μCT dataset, which cannot be attained by recent CT SR methods.

Here, we compare the MMSR loss with other loss terms proposed in previous methods, and discuss about the necessity of the MMSR loss. A relevant work named GAN-CIRCLE^29^ used adversarial loss, cycle-consistency loss, identity loss, and joint sparsifying transform loss to indirectly promote the consistency between input LR and output SR image. In contrast, our method imposes the MMSR loss to directly constrain input LR and output SR images have higher SSIM and pixel-wise similarity. In our newly built clinical CT−μCT dataset, LR and HR images have huge intensity and structural difference. Therefore, if we train SR methods without directly constraints between input LR and output SR images on our clinical CT−μCT dataset, the trained network tends to output SR images that is totally different from input LR images, such as results of pseudo-SR in Fig. 13. In contrast, using the MMSR loss, our method obtained satisfying qualitative and quantitative results. Another relevant network named CinCGAN^32^ uses modified identity loss and modified TV loss to ensure SR network’s output has higher pixel-wise similarity with input. However, CinCGAN only calculates the modified identity loss between input LR and output SR image. On the other hand, our method calculates MMSR loss from (1) input LR and output SR image and (2) HR image and corresponding synthesized LR image. Moreover, our MMSR loss is proposed based on two evaluation metrics: MSE and SSIM. Our method showed better performance than CinCGAN on MSE-based (PSNR) and SSIM-based evaluation metrics.

We can further differentiate our method from recent supervised and unsupervised CT SR methods. Recent supervised CT SR methods, such as ESRGAN for CT SR,^54^ require pairs of LR-HR images for training. In contrast, our method does not need any paired LR-HR images for training. Some image denoising methods could be applied in SR.^55^ GAN with network-in-network structure embed with skip connection naming deep convolutional generative adversarial network (DCSWGAN)^20^ was proved to be effective in CT image denoising. The generator of DCSWGAN consists of convolutional blocks, and each convolutional block consists of convolutional layer, bias, and leaky rectified linear unit, which is similar to our method’s generator $G_{1}$. The generator of DCSWGAN uses a cascade structure containing two subnetworks, one is a feature extraction network, the other is a reconstruction network. In contrast, our method only uses one network for SR. A disadvantage of DCSWGAN is that it still needs paired images for training. You et al. proposed an unsupervised SR method for CT and MRI images named GAN-CIRCLE,^29^^,^^56^ and further applied to bone micro structure reconstruction^57^ and brain MRI reconstruction.^58^ GAN-CIRCLE performed 2× SR (resolution of output SR image is two times of input LR image). On the other hand, we desire an 8× SR method which performs SR of clinical CT images to μCT scale. Our method achieved 8× SR (SR from 32×32  pixels to 256×256  pixels). Moreover, unsupervised SR methods such as CinCGAN^32^ and GAN-CIRCLE^29^ can only perform SR between images of the same modality (e.g., LR MRI images to HR MRI images); consequently, the LR and HR images do not have huge differences aside from resolution. Therefore, recent SR methods performed poorly on our clinical CT−μCT dataset, since our HR (μCT) and LR (clinical CT) images are from totally different modalities.

### 4.4 Analysis of Parameter Selection of Loss Terms

Here, we analyze the parameter selection of each loss term and discuss how assigning weights to each loss term leads to the best results. The overall loss function is composed of three terms: (1) SSIM loss, (2) downsample loss, and (3) upsample loss. Various combinations of loss terms lead to different quantitative results, as shown in Table 3. Table 3 shows that each loss function contributes to the final result. SSIM loss (containing two loss terms) brings the highest PSNR and SSIM score improvement. While the method is already equipped with SSIM loss, downsample loss and upsample loss can still improve PSNR and SSIM score slightly. Therefore, we believe that a higher weight of SSIM loss together with smaller weights of downsample loss and upsample loss brings the highest PSNR and SSIM score.

### 4.5 Effect of Downblocks in SR-CycleGAN

**Fig. 17:**
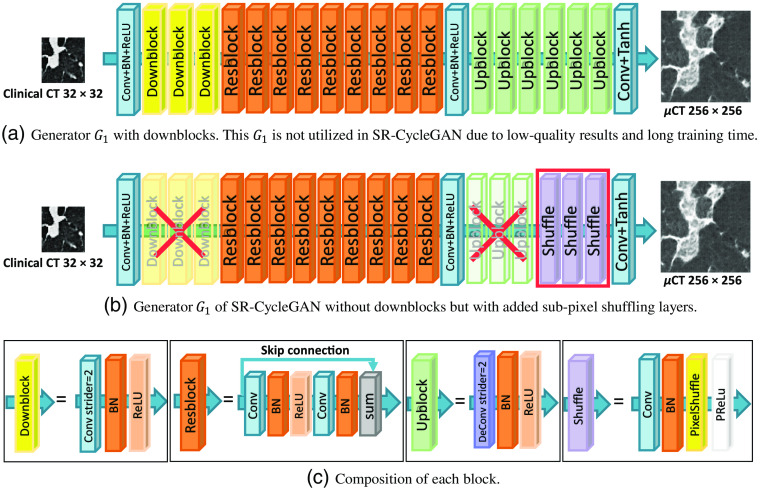
Two kinds of generator $G_{1}$ structures. We performed experiments on (a) $G_{1}$ with downblocks and (b) $G_{1}$ without downblocks but with added sub-pixel shuffling layers; (b) performed qualitatively and quantitatively better than (a). (c) Detailed compositions of each block.

We performed experiments to verify the effectiveness of removing downblocks and adding pixel-shuffling layers in generator $G_{1}$. As shown in Fig. 9, the SR results obtained by generator $G_{1}$ with downblocks and without pixel-shuffling layers [Fig. 17(a)] look blurred and noisy, while the SR results obtained by generator $G_{1}$ without downblocks and with sub-pixel shuffling layers [Fig. 17(b)] look clearer. This is because downblocks scale down the input images to a smaller size. Input images have 32×32  pixels; downblocks scale down the input images into feature maps of 4×4  pixels, and such small feature maps destroy spatial information in the input image. Furthermore, generator $G_{1}$ with downblocks [Fig. 17(a)] is deeper than generaor $G_{1}$ without downblocks [Fig. 17(b)]. Previous research affirmed that deeper stages of neural networks are more semantic but spatially coarser.^59^ Thus, the shape of essential anatomical structures such as the bronchus are likely to deform in the SR result, as shown in Fig. 9(b).

### 4.6 Effect of Reducing Computing Time Using Sub-Pixel Shuffling Layers

The sub-pixel shuffling layers were proved to shorten computing time, compared with upblocks.^36^ We replaced upblocks with sub-pixel shuffling layers in the proposed SR-CycleGAN. In Fig. 7, two kinds of network structures for generator $G_{1}$ are compared. The experimental results show that training time was significantly reduced from 491 to 353 s for training per epoch (2000 patches). For handling large-scale networks, such as CycleGAN, reducing computing time is an important issue. Introducing sub-pixel shuffling layers saved computing resources without loss of accuracy.

### 4.7 Difficulty of Quantitative Evaluation

In conventional SR methods, quantitative evaluation is typically conducted by comparing SR and HR image pairs. However, it is infeasible to obtain such pairs between clinical CT and μCT images, as mentioned in Sec. 1. To perform quantitative evaluation, we used downsampled μCT images instead of clinical CT images. We input the downsampled μCT image into trained generator $G_{1}$ and then obtained the SR result of downsampled μCT from $G_{1}$. Next, we compared the SR result with the original μCT images. We used PSNR to compare the SR image and the original μCT image. Since μCT images and clinical CT images have the same anatomical structures (bronchi and arteries), downsampled μCT images can simulate clinical CT images to a certain extent.

However, downsampled μCT images cannot simulate clinical CT images perfectly because the imaging conditions of μCT and clinical CT are different. For a specific tissues such as the bronchus in clinical CT, intensity is around −500 to 200 H.U. On the other hand, the intensity of the bronchus in μCT is around 6000 to 14,000 H.U. Furthermore, lung specimens for scanning μCT images are resected from part of the lung, so the μCT images of lung specimens do not contain anatomical information of the whole lung. Hence, we cannot simulate clinical CT perfectly by downsampling μCT images to the clinical CT scale. Therefore, in the future, we plan to propose a new evaluation matrix for the evaluation of SR-CycleGAN.

## 5 Conclusion and Future Work

We proposed an unsupervised SR method named SR-CycleGAN. We also proposed an innovative MMSR loss to ensure the SR image has similar anatomical structures and similar intensity distribution as the input LR image. Additionally, we improved the network structure to obtain both quantitatively and qualitatively better results. Experimental results demonstrate that our method is suitable for the SR of a lung’s clinical CT to the μCT scale, while conventional CycleGAN (without the proposed loss terms) outputs SR images with low qualitative and quantitative values.

Future work includes a more precise quantitative evaluation of our method. In addition, while our method focused on the SR of clinical CT to the μCT scale, it is not limited to the specific SR task of handling clinical CT for the lungs. Our method can also be applied to other SR tasks using medical images as a processing target. Therefore, applying our method to new data will also be among our future works. Since it is often difficult to register images from modalities with different resolutions, we believe that SR methods with training by unpaired LR and HR images will be essential and widely used in the near future.
